# Detection of Biomolecules Using Solid-State Nanopores Fabricated by Controlled Dielectric Breakdown

**DOI:** 10.3390/s24082420

**Published:** 2024-04-10

**Authors:** Peng Cheng, Candong Zhao, Qinjie Pan, Zijian Xiong, Qi Chen, Xiangshui Miao, Yuhui He

**Affiliations:** Hubei Yangtze Memory Laboratories, School of Integrated Circuit, Huazhong University of Science and Technology, Wuhan 430074, China

**Keywords:** nanopore, controlled dielectric breakdown, biomolecule detection

## Abstract

Nanopore sensor technology is widely used in biomolecular detection due to its advantages of low cost and easy operation. In a variety of nanopore manufacturing methods, controlled dielectric breakdown has the advantages of a simple manufacturing process and low cost under the premise of ensuring detection performance. In this paper, we have made enhancements to the applied pulses in controlled dielectric breakdown and utilized the improved dielectric breakdown technique to fabricate silicon nitride nanopores with diameters of 5 to 15 nm. Our improved fabrication method offers the advantage of precise control over the nanopore diameter (±0.4 nm) and enhances the symmetry of the nanopore. After fabrication, we performed electrical characterization on the nanopores, and the IV characteristics exhibited high linearity. Subsequently, we conducted detection experiments for DNA and protein using the prepared nanopores to assess the detection performance of the nanopores fabricated using our method. In addition, we also give a physical model of molecule translocation through the nanopores to give a reasonable explanation of the data processing results.

## 1. Introduction

In recent years, nanopore sensors have gained increasing prevalence in the detection of various biomolecules due to advantages such as low cost, rapid detection and brilliant performance [1,2]. Manrao et al. [3] achieved the discrimination of six DNA sequences with close readable regions ranging from 42 to 53 nucleotides in length using MspA nanopore combined with phi29 DNA polymerase. Zou et al. [4] utilized solid-state nanopores to detect translocation signals of two structurally distinct proteins and distinguish the two proteins based on differences in current blockade and dwell time distributions. Xi et al. [5] achieved a specific recognition of miRNAs by designing a nucleic acid-modified probe for α-hemolysin nanopores.

The principle of biomolecule detection by nanopore is illustrated in Figure S1. When a voltage is applied at both ends, negatively charged biomolecules will be driven by electric field and moves towards the positive electrode. When a biomolecule enters the nanopore, the ion current will decrease because the physical occupation of the biomolecule blocks the ion transport, and the current blockade formed during the translocation of biomolecules through the nanopore provides relevant structural information [6].

Nanopores are broadly categorized into two main types: biological nanopores derived from protein ion channels [7,8,9], and solid-state nanopores manufactured from materials such as silicon nitride [10,11,12,13], silicon dioxide [14,15,16], graphene [17,18,19], boron nitride [20,21] and molybdenum disulfide [22]. The solid-state nanopore has attracted much attention because of its advantages such as a controllable diameter, good stability and the potential for batch fabrication [6].

In recent years, various methods for fabricating solid-state nanopores have been developed [23]. To meet the requirements of biomolecular detection, it is essential to ensure that the diameter of the nanopore be slightly larger than the size of the biomolecule. Therefore, in order to meet the application demands of high-precision detection, it is imperative to develop methods for fabricating nanopores with ultra-small diameters. Focused ion beam (FIB) and focused electron beam (FEB) are two commonly used fabrication methods capable of meeting this criterion. Gierak et al. [24] utilized focused ion beam techniques to fabricate a series of nanoholes with an average diameter of 4.5 nm and a minimum diameter of 2.5 nm. Rigo et al. [25] prepared nanopores with diameters ranging from 0.28 nm to 1 nm by focused electron beam. However, they both exhibit high costs and operational complexities [6]. In contrast, controlled dielectric breakdown emerges as a highly convenient and cost-effective method for nanopore fabrication [26,27,28,29,30,31]. This technique requires only a device capable of generating controllable voltage/current pulses, a feature readily available in most laboratories. Furthermore, this method is also capable of producing nanopores with ultra-small diameters. For example, Yanagi et al. [31] employed this approach to fabricate nanopores with diameters ranging from sub-1 nm to 3 nm. 

In this study, we have redesigned the pulses applied during the dielectric breakdown process for the precise control of the diameter of the nanopores. Utilizing our improved controlled dielectric breakdown method, we can precisely manipulate the diameter of the nanopores (±0.4 nm) and enhance their symmetry, resulting in shapes closer to cylinders. This paper delineates the process of fabricating silicon nitride nanopores through our improved controlled dielectric breakdown and presents the results of biomolecule detection using the manufactured nanopores. We also give a reasonable analysis of the molecule translocation based on the detection results. Our results further confirm that nanopores fabricated by controlled dielectric breakdown are competent for the detection of biomolecules.

## 2. Materials and Methods

### 2.1. Nanopore Fabrication

**Figure 1:**
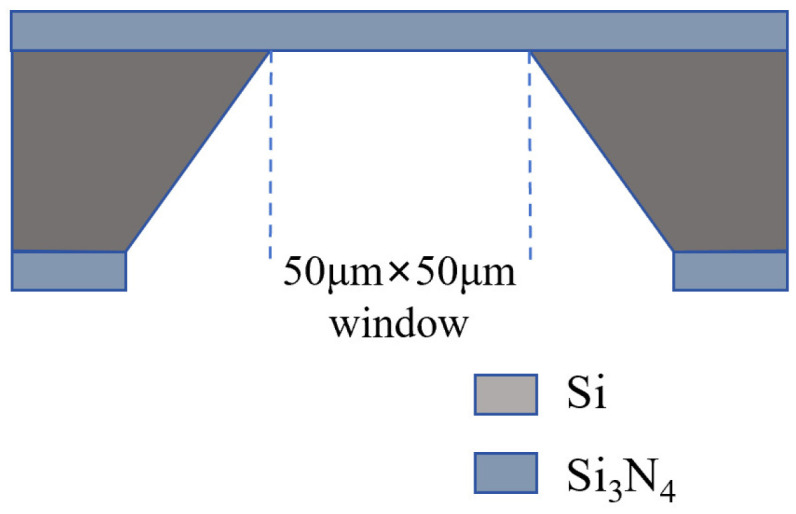
The structure of the Si_3_N_4_ membrane chip.

The Si_3_N_4_ membrane chip used for nanopore fabrication is a testing product provided by Shenzhen Jieyun Biotech Co., Ltd., Shenzhen, China, structurally similar to YWMEMS CleanSiN products. The structure of the Si_3_N_4_ membrane chip is illustrated in Figure 1, where a 15 nm thick silicon nitride film is positioned on the silicon support layer with a 50 μm × 50 μm window in the center. The specific manufacturing process is detailed as follows: a Si_3_N_4_ chip was clamped with the assistance of two rubber gaskets within the specially designed flowcell illustrated in Figure S2. The chip was securely fastened using screws. Ethanol was sequentially injected into the liquid chambers on both sides, ensuring the absence of residual air bubbles. Subsequently, the ethanol was drained, followed by several rinses with deionized water. Finally, the chip was washed multiple times with a 1 M KCl-TE solution (pH = 8). The KCl-TE solution was then introduced into both ends, preparing the chip for subsequent controlled dielectric breakdown pore formation operations.

The equipment employed for controlled dielectric breakdown was the Keithley 2450 digital source meter, which is controlled through LabVIEW2016 software on a computer to regulate its output and continuously monitor the current and voltage in the circuit. Two Ag/AgCl electrodes were inserted into the liquid chambers on either side of the flow cell, with the other ends connected to the source meter. Pore formation was conducted using a pre-programmed LabVIEW script, requiring simple adjustments to the initial voltage/current and step size. 

**Figure 2:**
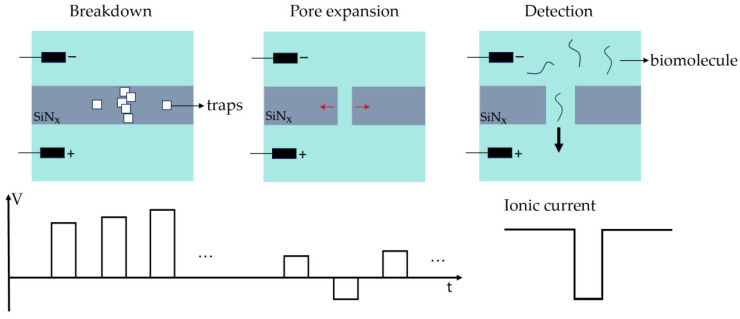
The nanopore fabrication process and the subsequent biomolecule detection process. The fabrication process is divided into two stages: high-voltage breakdown and low-voltage pore expansion. The detection process was based on the ion current blockade caused by the translocation of biomolecules.

We adopted a multi-step current pulse breakdown approach, applying a series of unipolar current pulses with an initial amplitude of 30 nA and a step size of 5 nA. Simultaneously, the LabVIEW program dynamically fits the estimated curve of the nanopore diameter over time based on the calculated electrical conductance of a cylindrical nanopore. The pulse was terminated when the curve suddenly approached a parallel rise along the *y*-axis, and the increase reached 0.5 nm, indicating the breakdown of the silicon nitride membrane. Subsequently, current pulses were halted and switched to bipolar voltage pulses with an initial amplitude of 3 V and a step size of 0.02 V. Figure 2 describes the nanopore fabrication process and the subsequent biomolecule detection process. Here the critical step was to switch to lower amplitude voltage pulses after breakdown occurs. The low amplitude of the voltage pulses facilitates a gradual increase in pore size, facilitating pore size control. The bipolar characteristic of the voltage pulses helps to enhance the symmetry of nanopores during pore expansion. As a result, nanopores with a cylindrical shape were formed, beneficial for the analysis of results from biomolecule translocation experiments.

### 2.2. Nanopore Characterization

For nanopores fabricated by controlled dielectric breakdown, it is exceedingly challenging to locate nanopores of ultra-small diameters under an electron microscope because they cannot be observed in situ like nanopores fabricated by FIB or TEM. As a result, only ion current characterization was performed for nanopores fabricated by our improved controlled dielectric breakdown method, enabling an estimation of their diameter and adequately meeting the analytical requirements. 

The Axon Digidata 1550B low-noise data acquisition system and Axopatch 200B patch-clamp amplifier (both manufactured by Molecular Devices, San Jose, CA, USA) were used to perform current characterization and the subsequent biomolecular detection of the fabricated silicon nitride nanopores. Following the fabrication of the Si_3_N_4_ nanopore, the chip was to be maintained within the flowcell, subsequently moved into a Faraday cage. Then the two Ag/AgCl electrodes situated in the respective chambers were to be connected to the Axopatch 200B patch-clamp. Utilizing the “Clampex10.4” software on the interconnected computer, the voltage applied to the chambers can be configured, and current readings can be acquired in correspondence to distinct voltage values. By changing the KCl concentrations and measuring the current at different voltages, a I–V curve for a fabricated nanopore under varying KCl solution concentrations was obtained. Subsequently, the conductance values of the nanopore at different KCl concentrations were derived from the I–V characteristics, enabling the construction of a relationship curve between nanopore conductance and KCl concentration. 

For cylindrical nanopores, the conductance can be calculated as follows [32]:

(1) $$G=e(\mu_{K}+\mu_{Cl})n_{KCl}{\left(4L/\pi d^{2}+1/d\right)}^{-1}+\pi d\sigma \mu_{K}/L$$

where, L is the length of the nanopore, d is the diameter of the nanopore, σ is the surface charge density of the nanopore wall and $\mu_{K}$ and $\mu_{Cl}$ are the mobility of K^+^ and Cl^−^, respectively. The second term of Equation (1) is caused by the surface charge of the pore wall, which dominates at low concentrations and is far smaller than the first term at high concentrations. Therefore, at 1 M KCl, we can ignore the second term and solve an approximation of d based on the conductance. Subsequently, according to the conductance of the nanopore when the concentration is extremely low and the second term in the equation is much higher than the first term, the value of the surface charge σ of the pore wall can be obtained according to Equation (1), which will be used for the following theoretical calculation.

### 2.3. Biomolecule Detection Experiment

The chosen DNA for the detection experiment is commonly used λ-DNA and the protein applied in this research is Recombinant human Caspase-9 protein from Biorbyt, both of which carry a negative charge on its surface under the experimental conditions with a pH of 8. By adding the biomolecule to the cis side (the chamber linked to the negative terminal) of the flowcell and applying a proper voltage across the liquid chambers, continuous monitoring of the current blockade signal could be observed over a specified period. Different voltages were applied at both ends to record the corresponding translocation signals of proteins, aiming to investigate the influence of voltage on the translocation of biomolecules through the nanopore. Furthermore, we investigated the influence of salt solution concentration on the translocation of biomolecules through the nanopore by change the KCl concentration. 

## 3. Results

### 3.1. Characterization Results of Nanopores

**Figure 3:**
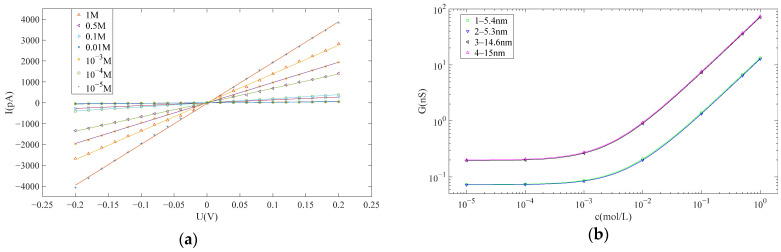
(**a**) The current–voltage curve of the nanopore at different KCl concentration. (**b**) The conductance–KCl concentration curve of the four nanopores.

In accordance with the methodology outlined in Section 2.2, I–V curves for nanopores at various concentrations could be obtained, enabling the estimate of their pore sizes. Subsequently, the pore sizes for the four nanopores utilized in the subsequent detection experiments were estimated as follows: 5.4 nm, 5.3 nm, 14.6 nm and 15.0 nm. The first two nanopores were employed for DNA detection, while the latter two were employed for protein detection. Figure 3a presents the I–V curves of the nanopore with a diameter of 5.4 nm at various concentrations, while Figure 3b illustrates the relationship between the conductivity of all four nanopores and the KCl concentration. Notably, the I–V curve exhibits excellent linearity, indicating a high level of cleanliness for the nanopore.

### 3.2. DNA Detection by Nanopore

#### 3.2.1. Individual Event Analysis

**Figure 4:**
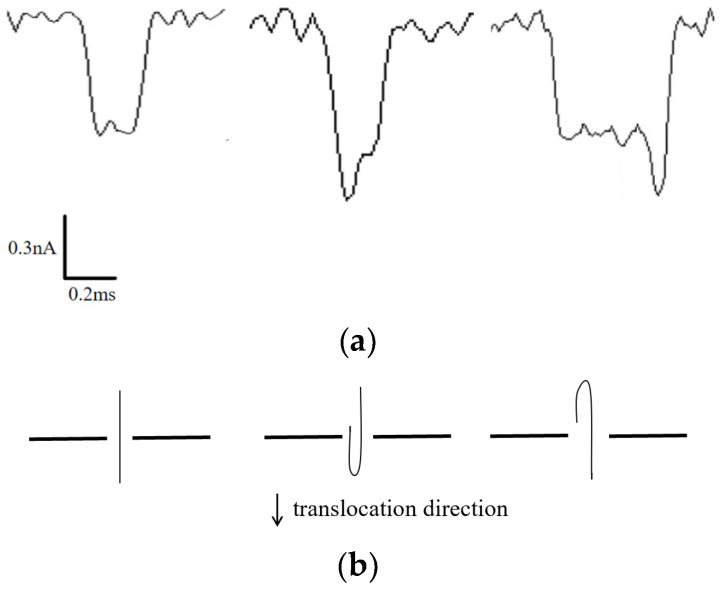
(**a**) DNA translocation signals of different types. (**b**) DNA translocation posture corresponding to different translocation signals.

Individual translocation events were isolated for detailed analysis, as depicted in Figure 4a. Distinctive shapes of the current blockade waveform reflect different translocation postures of the DNA molecules. If the blockade signal exhibits symmetrical characteristics, it suggests that the DNA chains translocate through the nanopore in a predominantly linear fashion. Conversely, if the blockade signal waveform displays step-like features, it indicates varying degrees of folding during the DNA translocation. Figure 4b illustrates three potential DNA translocation configurations corresponding to the three waveform patterns observed in Figure 4a. Further consideration of the amplitude and translocation duration of individual blocking signals allows for a more refined analysis of the translocation dynamics of the DNA molecules.

#### 3.2.2. Experiment of Different Voltages

**Figure 5:**
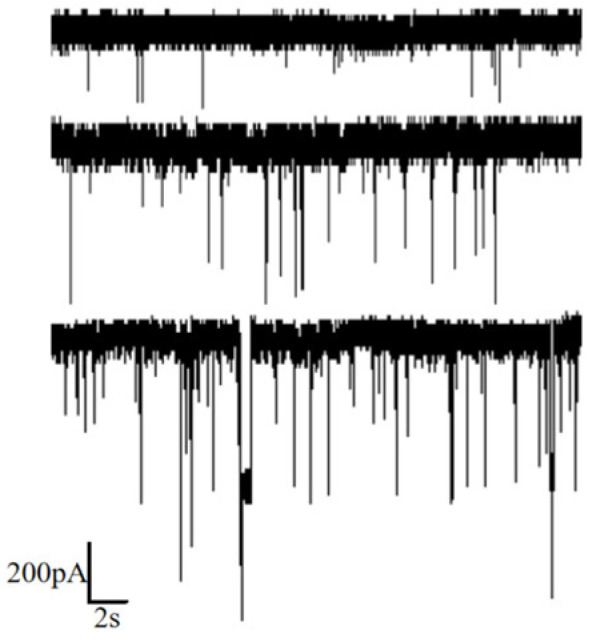
Ion current signal of DNA detection under different voltages (50 mV, 100 mV and 150 mV from top to bottom).

Voltages of 50 mV, 100 mV and 150 mV were applied, and the corresponding current signals were recorded, as illustrated in Figure 5. It is evident from the figure that higher applied voltages result in a larger amplitude of the corresponding current blockades. Additionally, the number of translocation events within the same time frame increased with higher voltages, indicating a higher capture rate. 

**Figure 6:**
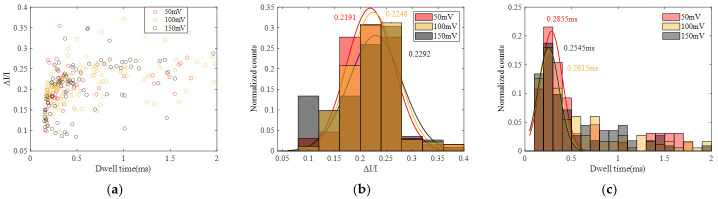
Statistics of DNA translocation at different voltage (50 mV, 100 mV and 150 mV). (**a**) Scatter diagram with ΔI/I and dwell time. (**b**) Histogram of ΔI/I. (**c**) Histogram of dwell time.

To analyze the distribution of current blockade amplitude and dwell time, the statistics of current blockade ratio (ΔI/I) and dwell time at 50 mV, 100 mV and 150 mV were calculated, as shown in Figure 6, and the statistics of the current blockade and dwell time are illustrated in Figure S3. Each distribution is fitted by a Gaussian curve. It can be seen that the peak current blockade increases as the voltage increases and the peak dwell time decreases as the voltage increases, while the difference in ΔI/I is not significant. As the voltage rises, the corresponding baseline current also increases, leading to larger current blockades caused by molecule occupation. Additionally, with increasing voltage, the force acting on DNA molecules due to the electric field becomes stronger, resulting in faster translocation and, consequently, a reduction in translocation time.

#### 3.2.3. Experiment of Different Salt Concentration

**Figure 7:**
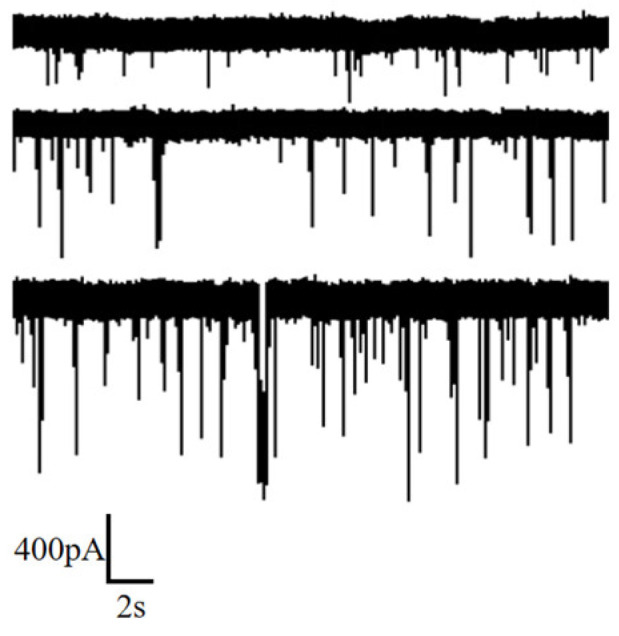
Ion current signal of DNA detection under different KCl concentrations (0.5 M, 0.75 M and 1 M from top to bottom).

Measurements were conducted under 0.5 M, 0.75 M and 1 M KCl solutions, applying a voltage of 100 mV. The obtained ion current signals are illustrated in Figure 7. It is observed that an increase in salt solution concentration enhances the capture rate of DNA by the nanopore. Additionally, higher concentrations correspond to a larger amplitude in the current blockade. 

**Figure 8:**
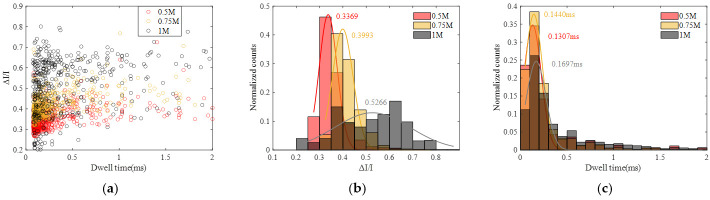
Statistic data of DNA translocation at different KCl concentration (0.5 M, 0.75 M and 1 M). (**a**) Scatter diagram with ΔI/I and dwell time. (**b**) Histogram of ΔI/I. (**c**) Histogram of dwell time.

Similarly, statistical analyses were performed, and the results are presented in Figure 8 and Figure S4. Analysis reveals that the peak current blockade and current blockade ratio increase with the rise in KCl concentration, while the peak dwell time exhibits an increment with increasing KCl concentration. The increase in current blockade can be readily understood; as the concentration of KCl rises, the resulting ionic current also increases, naturally leading to a larger current blockade. The increase in current blockade ratio indicates an increase in the spatial occupancy of DNA within the pore, which may be related to the effect of salt concentration on the surface charge of DNA. The elongation of dwell time can be attributed to the reduction in the negative charge carried by the DNA surface. With the increase in KCl concentration, the adsorption of K^+^ on the DNA surface reduces the net charge, diminishing the driving force from the electric field and resulting in a prolonged translocation time.

#### 3.2.4. Theoretical Analysis

Here, we establish a theoretical model of DNA translocation and calculate the current amplitude and dwell time of blockade signal generated by DNA translocation through the nanopore under given conditions under a reasonable approximation. Then we compare them with the experimental results, and analyze and interpret the experimental results.

It has long been found that there are surface charges on the inner wall of the nanopore, which significantly regulate the ionic current through the nanopore. The surface charges on the pore wall cause counterions and induce electric field. Let us take the KCl electrolyte as an example and continue the discussion. The induced potential in a nanopore can be described by the Poisson–Boltzmann equation:

(2) $$\nabla^{2}\phi =-\frac{\rho_{e}}{\varepsilon_{f}}=-\frac{e\sum_{i}z_{i}n_{i}}{\varepsilon_{f}}$$

(3) $$n_{i}=n_{i}^{0}\exp(-\frac{ez_{i}\phi }{kT})$$

In the above, ϕ is the induced potential, $\varepsilon_{f}$ is the permittivity of the fluid, $\rho_{e}$ is the net charge density, $z_{i}$ is the valence of the element, $n_{i}$ is the particle number density of the *i*-th ion, $n_{i}^{0}$ is particle number density of the *i*-th ion away from the nanopore. 

In the cylindrical coordinate, use the value of $z_{K}=+1,z_{Cl}=-1$ and $n_{i}^{0}=n_{0}$, we can get Equation (4) from Equations (2) and (3):

(4) $$\frac{1}{r}\frac{\partial }{\partial r}(r\frac{\partial \phi }{\partial r})=-\frac{e(n_{K}-n_{Cl})}{\varepsilon_{f}}=-\frac{en_{0}[\exp(-\frac{e\phi }{kT})-\exp(\frac{e\phi }{kT})]}{\varepsilon_{f}}$$

In the above, $n_{0}$ is the concentration of KCl solution away from the nanopore. Let $\bar{\phi }=e\phi /kT$, then Equation (4) can be rewritten as Equation (5):

(5) $$\frac{1}{r}\frac{\partial }{\partial r}(r\frac{\partial \bar{\phi }}{\partial r})=\frac{\sinh\bar{\phi }}{\lambda_{D}^{2}}$$

where $\lambda_{D}$ is the Debye length representing the thickness of the induced electric double layers:

(6) $$\lambda_{D}=\sqrt{\frac{\varepsilon_{f}kT}{2e^{2}n_{0}}}$$

In the following discussion, we assume that DNA passes through the nanopore along the central axis of the nanopore, and do not consider folding or multiple DNA strands. When DNA passes through the nanopore, on the surface of the DNA, we can get Equation (7) according to the Poisson equation:

(7) $$\nabla \cdot E={∯}_{d\Omega }E\cdot dS=\frac{\rho_{0}}{\varepsilon_{f}}$$

where E is the induced electric field and $\rho_{0}$ is the net charge density on the DNA surface. Let $\lambda_{DNA}$ represent the linear charge density of the double-stranded DNA molecule, a represent the radius of the DNA strand and $E_{r}$ represent the radial component of E. Equation (7) is simplified to Equation (8):

(8) $$\begin{array}{c} E_{r}\cdot 2\pi adl=\frac{\lambda_{DNA}dl}{\varepsilon_{f}}\\ E_{r}=\frac{\lambda_{DNA}}{2\pi a\varepsilon_{f}} \end{array}$$

According to the relationship between electric potential and electric field, Equation (9) can be easily obtained:

(9) $${\left.\frac{\partial \bar{\phi }}{\partial r}\right|}_{r=a}=-\frac{e\lambda_{DNA}}{2\pi a\varepsilon_{f}kT}$$

On the inner wall of the nanopore, the boundary conditions of the electric field can be obtained as follows:

(10) $$\begin{array}{l} D_{2r}-D_{1r}=\sigma_{w}\\ \varepsilon_{p}E_{2r}-\varepsilon_{f}E_{1r}=\sigma_{w}\\ {\left.\frac{\partial E}{\partial r}\right|}_{r=R}=E_{2r}-E_{1r}=\frac{\sigma_{w}}{\varepsilon_{p}-\varepsilon_{f}} \end{array}$$

In the above, $\sigma_{w}$ is the surface charge density of the Si_3_N_4_ wall; $\varepsilon_{p}$ is the permittivity of Si_3_N_4_; *R* is the radius of the nanopore; $D_{2r}$ and $D_{1r}$ are the radial components of the electric displacement vector when *r* = *R^+^* and *r = R^−^*; and $E_{2r}$ and $E_{1r}$ are the radial components of the electric field when *r = R^+^* and *r = R^−^*. Similarly, Equation (10) can be obtained according to the relationship between electric potential and electric field:

(11) $${\left.\frac{\partial \bar{\phi }}{\partial r}\right|}_{r=R}=\frac{e\sigma_{w}}{kT(\varepsilon_{p}-\varepsilon_{f})}$$

Then the value of $\bar{\phi }$ can be calculated by solving the differential Equation (5) under the boundary conditions of Equations (9) and (11).

The fluid flow can be described by the following Navier–Stokes equation:

(12) $$\left\{\begin{array}{c} \rho_{f}(\frac{\partial u}{\partial t}+u\cdot \nabla u)=-\nabla p+\mu \nabla^{2}u+f_{e}\\ \nabla \cdot u=0 \end{array}\right.$$

In the above, $\rho_{f}$ is the density of the fluid; u is the flow rate; p is the pressure; μ is the viscosity of the fluid; and $f_{e}$ is the electric body force exerted on the fluid by the local charges of the unbalanced ions:

(13) $$f_{e}=(-\nabla V)e\sum_{i}z_{i}n_{i}$$

where *V* is the voltage applied to two chambers.

It is assumed that the fluid flow meets the following conditions: (1) steady state transport (∂u/∂t=0); (2) translational invariance along pore axis (∂u/∂z=0); and (3) limited by the pore wall ($u_{x}=u_{y}=0$). By combining them with Equations (5), (6), (12) and (13), we can get the following equation:

(14) $$\frac{1}{r}\frac{\partial }{\partial r}(r\frac{\partial u_{z}}{\partial r})=\frac{2n_{0}eE_{z}}{\mu }\sinh\bar{\phi }$$

where $E_{z}$ is the electric field along the pore axis formed by the applied voltage, which can be calculated from the applied voltage according to Ohm’s law as follows:

(15) $$\left\{\begin{array}{l} E_{z}=\frac{V_{z}}{L}\cdot \frac{R_{pore}}{R_{pore}+2R_{acc}}\\ R_{acc}=\frac{\rho }{4R}\\ R_{pore}=\frac{\rho L}{\pi R^{2}} \end{array}\right.$$

where $R_{pore}$ is the resistance of the nanopore material and $R_{acc}$ is the access resistance between the nanopore and the chambers on both sides.

The flow rate of the fluid satisfies the following non-slip boundary conditions:

(16) $$\left\{\begin{array}{l} {\left.u_{z}\right|}_{r=a}=u_{DNA}\\ {\left.u_{z}\right|}_{r=R}=0 \end{array}\right.$$

where $u_{DNA}$ is the velocity of DNA.

We assume that the velocity of the DNA strand in the inner part of the nanopore remains roughly constant as it passes through the pore. Therefore, the driving force exerted on a small DNA segment of dl length should be offset by the viscous driving force in the liquid [31]:

(17) $$\begin{array}{c} df_{z}=-\lambda_{DNA}E_{z}dl+\mu {\left.\frac{\partial u_{z}}{\partial r}\right|}_{r=a}2\pi adl=0\\ {\left.\frac{\partial u_{z}}{\partial r}\right|}_{r=a}=-\frac{E_{z}\lambda_{DNA}}{2\pi a\mu } \end{array}$$

And by solving the differential Equation (14) under the boundary conditions of Equations (16) and (17), we can get the velocity of the fluid and the DNA. Then the dwell time of DNA can be calculated according to the length of the nanopore and the DNA strand.

Finally, we solve for the ionic current, as shown in Equation (18):

(18) $$I=\int_{a}^{R}rdr\int_{0}^{2\pi }d\theta \sum_{i}ez_{i}(n_{i}v_{z}+n_{i}\mu_{i}E_{z})$$

In the above, the first term in parenthesis $J_{a}^{i}=n_{i}v_{z}$ represents the advection current of the *i*-th ion, and the second term $J_{d}^{i}=n_{i}\mu_{i}E_{z}$ represents the drift current [33]. $\mu_{i}$ is the ionic mobility.

Based on the aforementioned formula, theoretical calculations can be conducted to determine the amplitude of current blockades and dwell time when DNA strands pass through nanopores. These theoretical results can then be compared with experimental data. Taking different DNA translocations at various concentrations as examples, we introduced KCl solution concentrations of 0.5 M, 0.75 M and 1 M into the theoretical model. The calculated translocation times were 0.1144 ms, 0.1291 ms and 0.1426 ms, respectively. In comparison, the Gaussian fit peak values of translocation times in Figure 7 at different concentrations were 0.1307 ms, 0.1440 ms and 0.1697 ms. The theoretical calculations align well with the experimental data, demonstrating the reasonableness of the theoretical model. The calculated current blockade amplitudes were 93.9 pA, 163.8 pA and 230 pA for the respective concentrations. These results closely resemble the amplitudes obtained by applying the classical current blockade formula $\Delta I\approx I_{open}{\left(R_{DNA}/R_{\mathrm{pore}}\right)}^{2}$ [34]. This suggests the validity of our model. However, it is worth noting that these results are significantly lower than the current blockades derived from the experimental data, which were 234.17 pA, 418.9 pA and 739.5 pA. We speculate that this discrepancy arises from the fact that when a single DNA strand translocates through the nanopore, other DNA strands may also be driven by the electric field and adsorb around the vicinity of the nanopore. This partial obstruction alters the ion transport path within the nanopore, effectively reducing its effective pore size, resulting in an increased value of $R_{DNA}/R_{\mathrm{pore}}$ and consequently, an increased amplitude of the blockade.

In conclusion, our theoretical model can reasonably approximate the process of DNA translocation through nanopores. It enables a meaningful theoretical analysis in conjunction with experimental data.

### 3.3. Protein Detection by Nanopore

Due to the complex spatial structure of proteins, unlike the linear structure of DNA, obtaining the direct translocation configuration of proteins is challenging. For more precise protein analysis, the separation and purification of proteins, unfolding them into peptide chains, are required before conducting nanopore detection. Here we primarily focus on the statistical distribution of current blockade signals of protein translocation.

#### 3.3.1. Experiment of Different Voltages

**Figure 9:**
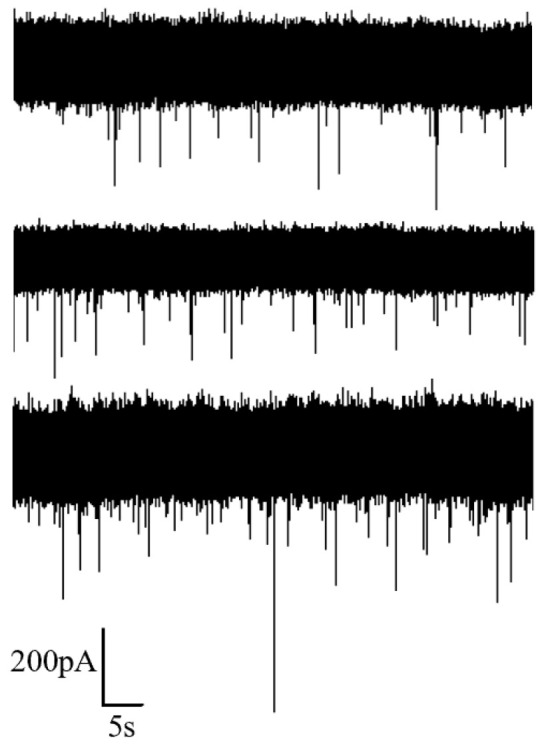
Ion current signal of protein detection under different voltages (100 mV, 120 mV, 140 mV from top to bottom).

Different voltages of 100 mV, 120 mV and 140 mV were applied at both ends to record the corresponding translocation signals of proteins, aiming to investigate the influence of voltage on the translocation of protein molecules through the nanopore. The recorded ionic current at different voltages is illustrated in Figure 9. It is evident from the figure that, with an increase in voltage, there is a certain degree of enhancement in the capture rate of proteins by the nanopore. However, compared to DNA, the capture rate for proteins is noticeably lower. Additionally, the amplitude of the blocking current in protein translocation signals exhibits less pronounced variations. Therefore, subsequent statistical analyses only adopt the relative current blockade, ΔI/I, as a descriptor for protein translocation.

**Figure 10:**
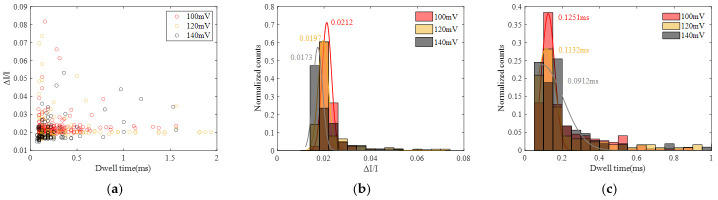
Statistic data of protein translocation at different voltages (100 mV, 120 mV and 140 mV). (**a**) Scatter diagram with ΔI/I and dwell time. (**b**) Histogram of ΔI/I. (**c**) Histogram of dwell time.

The results of the statistical analysis are presented in Figure 10, from which we can observe that the Gaussian fitting peak values of ΔI/I and dwell time decrease with increasing voltage. The reduction in dwell time can be attributed to the intensified electric field resulting from the higher applied voltage. The decrease in ΔI/I indicates a reduction in the spatial occupancy of the protein during translocation through the nanopore. As the voltage increases, the stretching effect of the voltage on the peptide chain leads to an increased unfolding degree [35], causing a decrease in the spatial occupancy of the protein during translocation and consequently a reduction in the value of ΔI/I.

#### 3.3.2. Experiment of Different Salt Concentration

**Figure 11:**
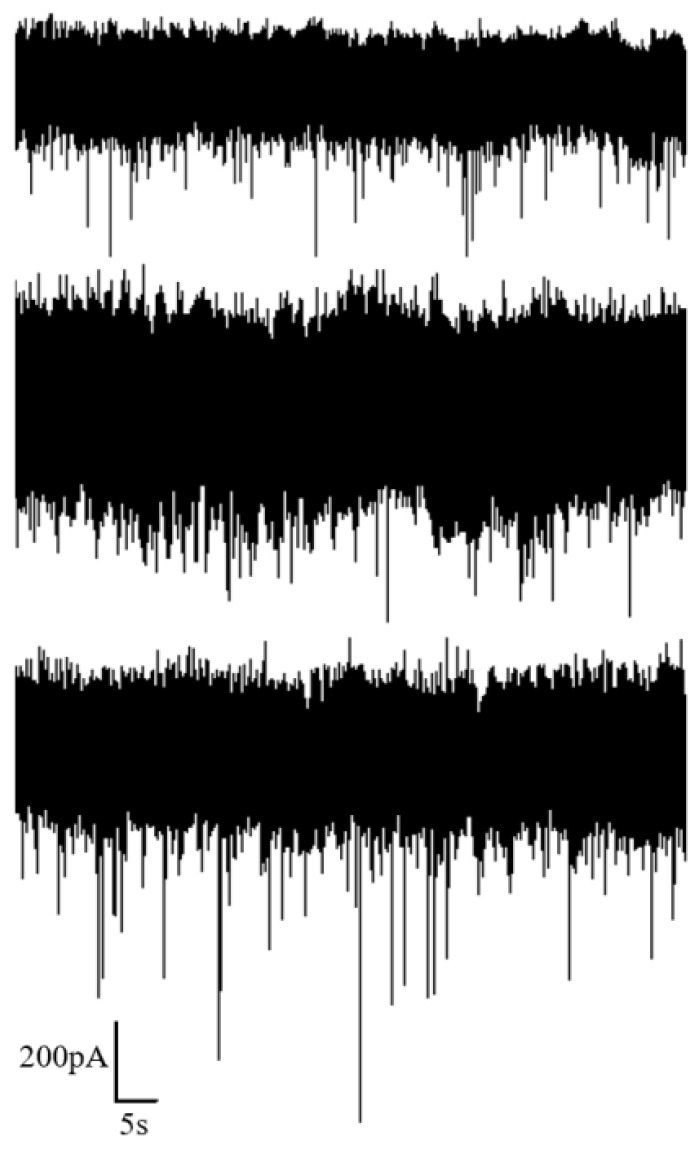
Ion current signal of protein detection under different KCl concentrations (1 M, 2 M and 5 M from top to bottom).

Maintaining the applied voltage at 100 mV, we varied the salt solution concentration on both sides of the liquid chambers to 1 M, 2 M and 5 M, respectively, and measured the translocation signals of proteins, as illustrated in Figure 11. 

**Figure 12:**
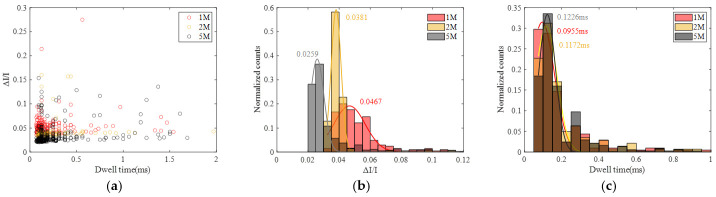
Statistic data for protein translocation at different KCl concentrations (1 M, 2 M and 5 M). (**a**) Scatter diagram with ΔI/I and dwell time. (**b**) Histogram of ΔI/I. (**c**) Histogram of dwell time.

Subsequently, statistical analysis was performed, and the results are summarized in Figure 12. From the figure, it is apparent that the Gaussian fitting peak value of ΔI/I decreases with increasing concentration, while the Gaussian fitting peak value of translocation time increases with concentration. Similar to the behavior observed in DNA translocation, the variation in translocation time can be explained by the reduction in the effective net charge on the molecular surface as the salt solution concentration increases, leading to a decrease in the driving force from the electric field and an elongation of the translocation time. The change in ΔI/I with concentration may be attributed to a reduction in the size of the protein with increasing salt concentration, resulting in a relatively smaller spatial occupancy. There are reports in the literature indicating a decrease in the gyration radius of proteins with increasing salt solution concentration [36], supporting the validity of the aforementioned speculation.

## 4. Conclusions

The presented article describes our improved controlled dielectric breakdown method to precisely fabricate nanopores with diameters below 15 nm and demonstrate the biomolecule detection performance using the fabricated nanopores. The deviation of the diameters of prepared nanopores from the set value are within 0.4 nm, demonstrating the precise control of pore size resulted from our fabrication method. In addition, the I–V curve of the prepared nanopores has high linearity, exhibiting the high quality of the nanopores. More importantly, the nanopores prepared using our approach provide biomolecule detection results comparable to those recently reported for nanopores fabricated by controlled dielectric breakdown [37]. The achieved sensing performance indicates that our improved controlled dielectric breakdown method is competitive. 

Our future work will focus on applying our improved controlled dielectric breakdown method to the fabrication of two-dimensional materials nanopores to obtain ultrathin nanopores with precise diameters. This advancement facilitates attaining nanopores with higher detection resolution at a low cost.

## Data Availability

Data are contained within the article.

## Supplementary Materials

The following supporting information can be downloaded at: https://www.mdpi.com/article/10.3390/s24082420/s1, Figure S1: The principle of biomolecule detection by nanopores; Figure S2: (a) The structure diagram of the flowcell (b) The flowcell used in the experiment; Figure S3: Statistics of DNA translocation at different voltage (50 mV, 100 mV, 150 mV). (a) scatter diagram with current blockade and dwell time (b) histogram of current blockade; Figure S4: Statistic data of DNA translocation at different KCl concentration (0.5 M, 0.75 M, 1 M). (a) scatter diagram with current blockade and dwell time (b) histogram of current blockade.
