# Supplementary material for: Detection of Biomolecules Using Solid-State Nanopores Fabricated by Controlled Dielectric Breakdown
Source: Sensors (Basel). 2024 Apr 10;24(8):2420. doi: 10.3390/s24082420 (PMC11053845; doi:10.3390/s24082420)
Supplement: Supplementary file 1 [file sensors-24-02420-s001.zip › sensors-2894750-supplementary.pdf]

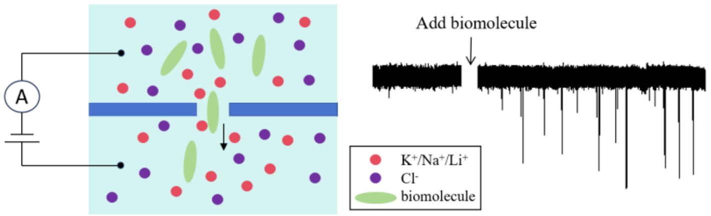

Figure S1. The principle of biomolecule detection by nanopores.

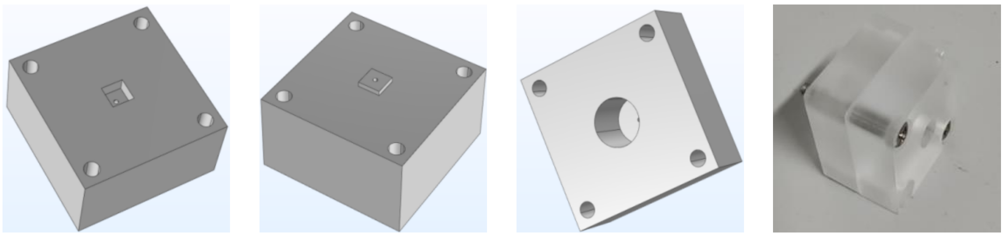

(**a**) (**b**)

Figure S2. (**a**) The structure diagram of the flowcell (**b**) The flowcell used in the experiment.

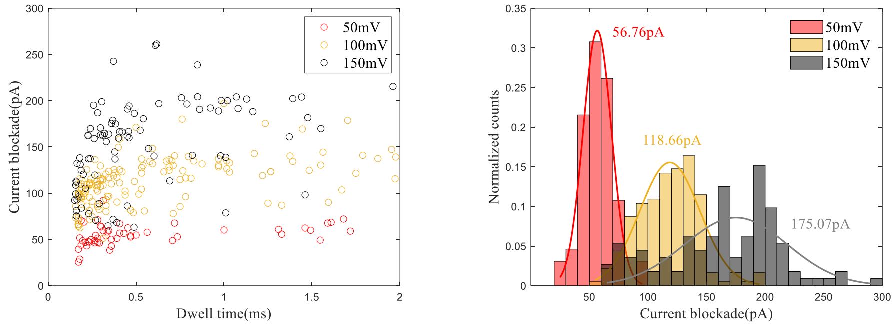

(**a**) (**b**)

Figure S3. Statistics of DNA translocation at different voltage(50mV,100mV,150mV). (**a**) scatter diagram with current blockade and dwell time (**b**) histogram of current blockade.

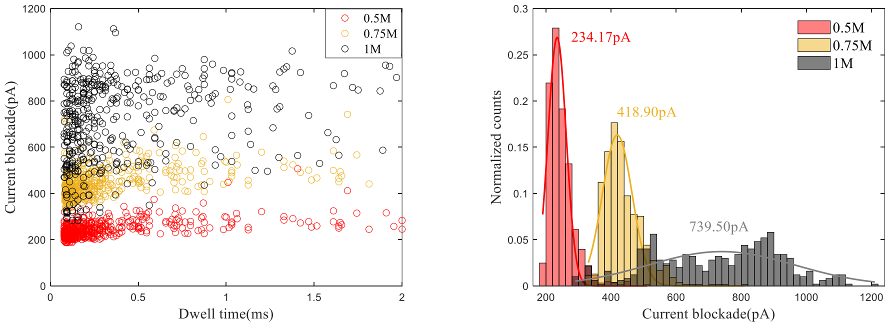

(**a**) (**b**)

Figure S4. Statistic data of DNA translocation at different KCl concentration (0.5M,0.75M,1M). (**a**) scatter diagram with current blockade and dwell time (**b**) histogram of current blockade.
